# From Blocking Shots to Blocking GI Transit, This Professional Basketball Player Does It All: A Case Report on Small Bowel Obstruction

**DOI:** 10.1155/2021/5534945

**Published:** 2021-04-01

**Authors:** Emily L. DeMaio, Richard C. Jarvis, Jessica A. Cohen, Courtney N. Gleason

**Affiliations:** Investigation Performed at the Emory Clinic, Department of Orthopaedic Surgery, Emory University, Atlanta, Georgia

## Abstract

Small bowel obstructions (SBO) are a commonly encountered diagnosis within emergency departments. Typically, these patients have evident risk factors including, but not limited to, prior abdominal surgery, personal or family history of gastrointestinal disorders, femoral and inguinal hernias, or neoplasm. In this case, we describe an SBO in a female, professional athlete whose swift, severe symptom onset, rapid resolution with conservative treatment, lack of identifiable risk factors, and prompt return to high level competition without recurrence are certainly unique. A female professional basketball player in her mid-20's with no past medical history presented with a seven-hour history of worsening abdominal pain beginning in the epigastric region and migrating to the right lower quadrant. Physical exam did not reveal abdominal distension, tympany to percussion, or high-pitched bowel sounds. Initial differential diagnosis included appendicitis, ruptured ectopic pregnancy, and other genitourinary pathology. Computed tomography with contrast revealed distended loops of small bowel with wall thickening, enhancement, and decompressed loops of bowel distally, consistent with an SBO. Symptoms resolved after 24 hours with conservative treatment, including decompression with a nasogastric tube. The athlete returned to full participation five days after initial presentation without recurrence of symptoms. Outpatient gastroenterology workup was negative for predisposing conditions. This presentation is rare in the absence of bowel pathology, family history, or prior abdominal surgery. Perhaps, her profession as an athlete, with frequent air travel and extensive exercise, may have contributed to this unique presentation. This case report should serve as a reminder to all providers that SBOs can occur in young, active patients devoid of risk factors. Even in the absence of typical signs on physical examination, providers should use imaging as adjuncts based on their clinical gestalt and utilize conservative management, when appropriate, to maximize chances of recovery with minimal morbidity.

## 1. Introduction

Small bowel obstructions (SBO) occur when a functional or mechanical blockade impairs the transit of chyme, and later air, through the intestinal tract [1, 2]. There are many known risk factors that contribute to the development of an SBO, which include, but are not limited to, prior abdominal surgery, personal or family history of inflammatory bowel disease, personal history of neoplasm, femoral and inguinal hernias, or gastrointestinal motility disorders [3]. In the absence of these risk factors, idiopathic SBOs rarely occur in the young, athletic population, possibly because of regular exercise habits. Moderate exercise has been proven to accelerate transit of chyme, feces, and gas within the intestines, thereby reducing the likelihood of SBO [4, 5]. This case is unique in that the patient's presentation was more typical of appendicitis or a gynecologic pathology; thus, SBO was not suspected until computed tomography (CT) revealed a transition point and bowel wall edema [6]. Given that this diagnosis is so uncommon in this population, there is a paucity of data stating when it is safe to return to athletic competition after SBO. Therefore, the medical team used the athlete's symptoms as the guide in return to play decisions, and she was able to return to full participation five days after presentation without recurrence of symptoms.

## 2. Case Presentation

A female professional basketball player in her mid-20's presented to the Emergency Department (ED) with a seven-hour history of worsening, sharp abdominal pain. Her pain, reported 7 out of 10 on the numeric rating scale (NRS) and exacerbated by movement with no alleviating factors, began in the epigastric region and migrated to the right lower quadrant. The patient experienced worsening nausea and emesis in the ED, warranting administration of 4 milligrams (mg) of intravenous (IV) ondansetron (GlaxoSmithKline, Brentford, UK) and infusion of a one liter (*L*) lactated ringer bolus. Review of systems was negative for diarrhea, constipation, fever, chills, dysuria, or vaginal discharge. The patient had only consumed self-prepared food for the past day and had no recent changes to her diet, sick contacts, or recent illnesses. She had never experienced pain like this previously. Her housemates and close contacts had no similar symptoms. The patient reported a low likelihood of pregnancy, and her last menstrual period was within the last month. She also reported no personal or family history of gastrointestinal disorders, prior pregnancies, or abdominal surgery. At the time of presentation, she denied any prior or current use of medications, alcohol, tobacco, or recreational drugs.

The patient's initial vital signs were a temperature of 36.8 degree Celsius (°C), blood pressure of 131/65 millimeters of mercury (mm Hg), heart rate of 60 beats per minute (bpm), respiratory rate of 16 per minute, and an oxygen saturation of 99% on room air. She was in apparent discomfort, clutching her abdomen. Her physical exam revealed a nondistended abdomen with normal bowel sounds in all four quadrants. Tenderness to light and deep palpation in the right lower quadrant was apparent, but there was no guarding, rebound, or tympany to percussion on initial examination.

**Figure 1:**
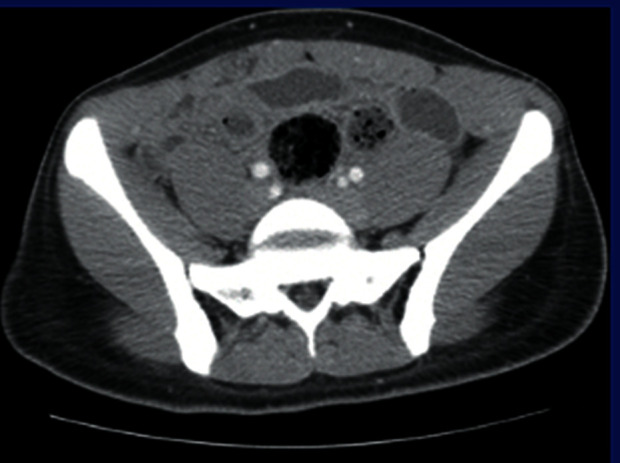
CT of the abdomen and pelvis with contrast showing distended loops of small bowel with wall thickening, enhancement, decompressed loops of bowel distally, and fecalization of small bowel.

**Figure 2:**
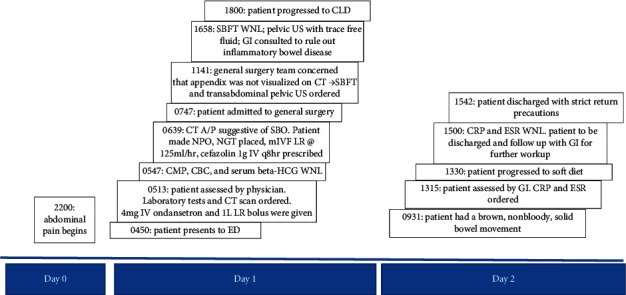
Timeline of symptom onset through hospital discharge. Day 0 represents symptom onset. Each event on timeline has time of occurrence listed in military time. SBFT, small bowel follow-through; WNL, within normal limits; US, ultrasound; GI, gastrointestinal; CT, computed tomography; A/P, abdomen and pelvis; SBO, small bowel obstruction; NPO, nil per os; NGT, nasogastric tube; mIVF, maintenance intravenous fluids; LR, lactated ringers solution; CMP, comprehensive metabolic panel; CBC, complete blood count; beta-HCG, human chorionic gonadotropin; ED, emergency department; CLD, clear liquid diet; CRP, C-reactive protein; ESR, erythrocyte sedimentation rate.

Initial workup included a comprehensive metabolic panel (CMP), complete blood count (CBC), and lipase, which were all within normal limits. A serum pregnancy test was negative. Urinalysis (UA) was only significant for trace ketones. CT of the abdomen and pelvis with IV contrast revealed distended loops of small bowel with wall thickening, enhancement, and decompressed loops of bowel distally. Fecalization of the small bowel was also apparent (Figure 1). The preliminary diagnosis was an acute SBO. Within the ED, conservative treatment was initiated including administration of cefazolin (GlaxoSmithKline, Brentford, UK) 1 gram (g) IV every 8 hours, maintenance of IV fluids at a rate of 125 milliliters per hour (mL/hr), placement of a nasogastric tube, and initiation of nil per os (NPO). The patient was admitted to the general surgery service. The timeline of events is illustrated in Figure 2.

**Figure 3:**
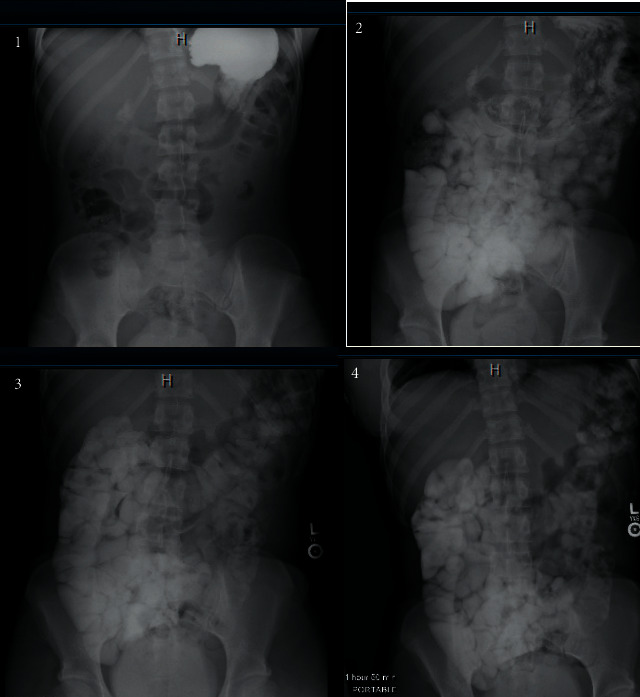
Upper gastrointestinal study with small bowel follow-through revealing no acute obstruction (radiographic images taken in series and are exhibited chronologically from 1–4.).

**Figure 4:**
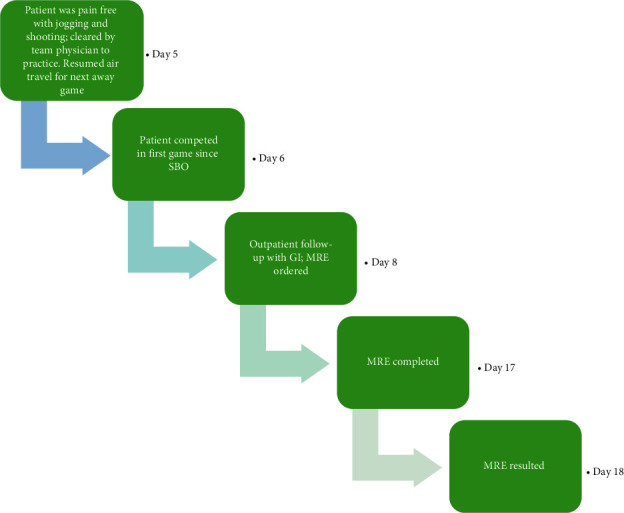
Chronologic depiction of return to play (RTP) and outpatient follow-up with days calculated since the time of symptom onset defined as day 0. SBO, small bowel obstruction; GI, gastroenterology; MRE, magnetic resonance enterography.

The general surgery team was concerned for a concurrent process, specifically appendicitis, because the appendix was not well evaluated on CT. Additionally, they wanted to assess the severity of the SBO; thus, a transabdominal pelvic ultrasound (US) and a small bowel follow-through (SBFT) were ordered, respectively. The US was only significant for trace free fluid in the Pouch of Douglas, and the SBFT showed resolution of the SBO at 12 hours after admission **(**Figure 3**).** At this point, a clear liquid diet was added, and gastroenterology (GI) was consulted to rule out inflammatory bowel disease (IBD) as the cause of the acute SBO which resolved rapidly. The GI consultants progressed the patient to a soft diet since she had a nonbloody, well-formed bowel movement and ordered a serum C-reactive protein (CRP) level and erythrocyte sedimentation rate (ESR), both of which were within normal limits. Thus, it was decided to follow up with GI as an outpatient for further workup. At the time of discharge, the patient was pain free, tolerating regular diet, and passing stool and flatus. At four days after presentation, the athlete's team physician allowed her to resume light jogging and shooting, followed by a full practice with her teammates later in the day. That same day, she flew with her teammates to the city of their next game. At five days after presentation, the athlete competed in a game and remained symptom-free. The patient was seen by GI seven days after presentation at which point magnetic resonance enterography (MRE) was ordered **(**Figure 4). The MRE showed no dilation, thickening, or enhancement of the bowel. These findings, coupled with her prior normal CRP and ESR, resulted in low suspicion for an underlying diagnosis of IBD, which would have been a potential predisposing condition for SBO. No other risk factors for SBO were ever identified so the patient's etiology remains idiopathic. Now, one year later, the patient remains without recurrence.

## 3. Discussion

Annually, there are 579–654 SBOs per 100,000 people, leading to over 300,000 surgeries in the United States [7]. However, it is atypical for a young, healthy, elite female athlete without a history of abdominal surgery or IBD to present with an SBO. In this population, abdominal pain is more frequently attributed to appendicitis, ovarian torsion, acute gastroenteritis, or IBD [8].

In the “virgin abdomen, ” defined as having no prior abdominal surgery, Beardsley et al. found adhesions to be the primary cause of SBO when diagnosed by CT and confirmed with laparoscopy; however, in this cohort, CT was only 52.9% accurate, and patients were subjected to unnecessary laparoscopy [9]. Furthermore, Colon et al. reported that presence of a transition zone on CT does not correlate with the need for surgery [10]. Therefore, the literature supports the decision to care for this patient nonoperatively given her stable appearance, reassuring vital signs, inherent risks of surgical intervention, and high likelihood of the SBO resolving with conservative therapy [2]. Despite the inherent limitations of imaging, CT remains the gold standard for workup of presumed SBO since definitive diagnosis with direct visualization via laparoscopy confers the common surgical risks of bleeding, infection, and damage to surrounding structures [7]. Because this patient lacked the classical findings of abdominal distension, tympany to percussion, and high-pitched bowel sounds, the diagnosis could have been missed if diagnostic imaging had not been pursued [2].

Despite previous reports that 13% of SBOs do not have identifiable etiology [11], it is important to be aware of unusual causes of SBO which may not be considered in the initial differential diagnosis and may require specific work up [12]. Bezoars, or concretions, are undigested material within the alimentary tract which can cause an SBO resulting in nonspecific symptoms similar to those appreciated in this case [13]. There are multiple types of bezoars, including: food bolus bezoars, phytobezoars, and trichobezoars [14]. Food bolus bezoars can stem from simple origins such as incomplete mastication, rapid deglutition, and consuming large nuts or pits [15]. Perhaps, an athlete in a rush may eat quickly and inadvertently increase the risk of a food bolus bezoar. Phytobezoars are primarily composed of undigestible plant fibers and seeds, and as such, a vegetarian diet may increase the patient's risk [16]. Trichobezoars are composed of undigested hair and are most frequently associated with trichotillomania with trichophagia. Trichobezoars can be seen in this patient's age group and should be considered as a possible etiology of SBO [17]. An extremely thorough clinician would be required to unmask these potential SBO etiologies via in-depth history and exam to uncover eating habits and areas of alopecia; however, in the ED setting, these questions are unlikely to arise. Furthermore, in a retrospective case series, CT scan was only 47% effective in identifying bezoars, so imaging alone cannot be depended upon to reach the diagnosis [13]. Thus, a high index of suspicion, coupled with history, physical, and diagnostic testing, is necessary to tailor treatment given that few bezoars will pass spontaneously and instead require endoscopic or open surgery [15, 18].

Intense physical activity can be another uncommon etiology of SBO. As aforementioned, studies suggest an inverse relationship exists between exercise and gastrointestinal disease with mild to moderate activity [5]. Despite this suggestion, de Oliveira and Burini caution that strenuous exercise coupled with dehydration exacerbates the risk of gastrointestinal symptoms [4]. Additionally, van Nieuwenhoven et al. hypothesized that running decreases small bowel transit time and blood flow, thereby increasing SBO risk [19]. Therefore, exercise serves both as a preventive measure for long-term GI disease, but in strenuous conditions, may potentiate acute illness.

The list of uncommon etiologies continues with researchers such as Massalou et al., suggesting that even recent air travel may contribute to SBO due to the threat barometric changes pose to hollow viscera. This likens the risk of SBO to the risk of deep vein thrombosis and pneumothorax associated with flight [20]. Although many of the aforementioned etiologies are speculative, careful consideration and awareness of the possibilities helps physicians to make sound medical decisions and tailor the treatment plan to the patient's unique presentation and risk factors.

Though definitive diagnosis of an SBO necessitates direct visualization via laparoscopy, history, physical examination, and CT were sufficient to suggest the diagnosis of an SBO in this case. Conservative management was successful, thereby avoiding unnecessary surgical risks. Furthermore, the patient was able to return to play more quickly because there was no surgical intervention. Given her comprehensive outpatient workup which failed to reveal any predisposing factors for her SBO, the exact etiology of the SBO remains unknown. This case highlights the importance of clinical acumen and utilization of adjunctive tests, specifically imaging, to render diagnoses and provide treatment suited to the patient's lifestyle. It also illustrates diagnostic challenges that may come from intrinsic difficulties of patient history taking. In order to identify a rare etiology for an SBO, a physician must be aware of the numerous possibilities and explore them thoroughly with the patient. Furthermore, this patient's case is unique in that she experienced full resolution of the SBO within twelve hours and returned to elite competition five days after initial presentation, despite the absence of clear recommendations within the literature regarding the timing of safe to return to play after an acute SBO.

## Data Availability

For further questions related to the content of this case report, the readers can contact the corresponding author.

## References

[1] Catena F. (2019). Bowel obstruction: a narrative review for all physicians. *World Journal of Emergency Surgery*.

[2] Jackson P., Vigiola Cruz M. (2018). Intestinal obstruction: evaluation and management. *American Family Physician*.

[3] Miller G., Boman J., Shrier I., Gordon P. H. (2000). Etiology of small bowel obstruction. *The American Journal of Surgery*.

[4] De Oliveira E. P., Burini R. C. (2009). The impact of physical exercise on the gastrointestinal tract. *Current Opinion in Clinical Nutrition & Metabolic Care*.

[5] Casey E., Mistry D. J., Macknight J. M. (2005). Training room management of medical conditions: sports gastroenterology. *Clinics in Sports Medicine*.

[6] Silva A. C., Pimenta M., Guimaraes L. S. (2009). Small bowel obstruction: what to look for. *Radio Graphics*.

[7] Kelly A. (2018). *Evidence-based Emergency Imaging: Optimizing Diagnostic Imaging of Patients in the Emergency Care Setting*.

[8] Hatipoglu S., Hatipoglu F., Abdullayev R. (2014). Acute right lower abdominal pain in women of reproductive age: clinical clues. *World Journal of Gastroenterology*.

[9] Beardsley C., Furtado R., Mosse C. (2014). Small bowel obstruction in the virgin abdomen: the need for a mandatory laparotomy explored. *The American Journal of Surgery*.

[10] Colon M. J., Telem D. A., Wong D., Divino C. M. (2010). The relevance of transition zones on computed tomography in the management of small bowel obstruction. *Surgery*.

[11] Ghosheh B., Salameh J. R. (2007). Laparoscopic approach to acute small bowel obstruction: review of 1061 cases. *Surgical Endoscopy*.

[12] Shatnawi N. J., Bani-Hani K. E. (2005). Unusual causes of mechanical small bowel obstruction. *Saudi Medical Journal*.

[13] Oh S. H., Namgung H., Park M. H., Park D.-G. (2012). Bezoar-induced small bowel obstruction. *Journal of the Korean Society of Coloproctology*.

[14] Eng K., Kay M. (2012). Gastrointestinal bezoars: history and current treatment paradigms. *Gastroenterology & Hepatology*.

[15] Lohn J. W. G., Austin R. C. T., Winslet M. C. (2000). Unusual causes of small-bowel obstruction. *Journal of the Royal Society of Medicine*.

[16] Chisholm E. M., Leong H. T, Chung S. C, Li A. K (1992). Phytobezoar: an uncommon cause of small bowel obstruction. *Annals of the Royal College of Surgeons of England*.

[17] Grant J. E., Odlaug B. L. (2008). Clinical characteristics of trichotillomania with trichophagia. *Comprehensive Psychiatry*.

[18] Dikicier E. (2015). Intestinal obstruction due to phytobezoars: an update. *World Journal of Clinical Cases*.

[19] Van Nieuwenhoven M. A., Brouns F., Brummer R.-J. M. (2004). Gastrointestinal profile of symptomatic athletes at rest and during physical exercise. *European Journal of Applied Physiology*.

[20] Massalou D., Fournier M., Salucki B., Baqué P. (2013). Small bowel obstruction secondary to transport aircraft: coincidence or reality?. *Clinics and Research in Hepatology and Gastroenterology*.

